# Managing Sophisticated Fraud in Online Research

**DOI:** 10.1001/jamanetworkopen.2024.60168

**Published:** 2025-02-27

**Authors:** Rebecca S. Mozaffarian, Jasmine M. Norris, Erica L. Kenney

**Affiliations:** 1 Department of Nutrition, Harvard TH Chan School of Public Health, Boston, Massachusetts; 2 Community Servings, Jamaica Plain, Massachusetts; 3 Department of Social and Behavioral Sciences, Harvard TH Chan School of Public Health, Boston, Massachusetts

## Abstract

This cross-sectional study examines fraudulent responses in an online study and shares strategies for preventing and detecting online recruitment fraud.

## Introduction

Researchers increasingly conduct online surveys or experiments.^1^ Unfortunately, online approaches can attract fraudulent respondents from individuals who are ineligible, but respond to distort results or obtain study incentives.^1,2,3,4,5^ This can be difficult to detect.^2,4^ We aim to share lessons we have learned from a recent online study to support other researchers.

## Methods

This cross-sectional study followed the Strengthening the Reporting of Observational Studies in Epidemiology (STROBE) reporting guideline and was approved by the Harvard Chan School of Public Health institutional review board. We obtained verbal consent from participants. We conducted a cross-sectional study measuring children’s exposure to advertising on mobile devices from March 2022 to April 2023. Originally, we attempted to enroll parents or guardians from Philadelphia, Pennsylvania, and Baltimore, Maryland. Study procedures involved parents or guardians completing an intake call and sending screenshots of children’s device battery screens; participants were offered incentives up to $70.

For initial recruitment from March to May 2022, we posted digital flyers describing eligibility criteria and the study incentive on an online classifieds site and public social media parenting groups in Philadelphia and Baltimore and received hundreds of eligibility screener responses. After enrolling several participants, we realized they had misrepresented themselves—some sent device data that was clearly from an adult and others were aggressive with staff about incentives. We unpublished the eligibility screener and flyers and reviewed our data and existing literature to identify indicators of fraud and best practices for mitigation to develop a prevention plan moving forward (eTable 1 in Supplement 1).

We next moved recruitment to Massachusetts (the study team’s location) to more easily conduct local outreach. We removed specific eligibility criteria from flyers and revised the eligibility screener to make the correct answers to eligibility criteria less obvious (eTable 2 in Supplement 1). Beginning June 2022, we posted the revised flyers in childcare programs and other community settings throughout Massachusetts. Recruitment was nondigital with 2 exceptions: (1) a childcare worker (who was trying to help us) posted about the study on her public social media page (August 2022); and (2) we used targeted social media advertisements that were supposed to reach only Massachusetts parents and guardians (October 2022).

We calculated the proportion of eligible, ineligible, or fraudulent eligibility screener responses for 2-week intervals across the study. Responses were coded as fraudulent if they met 2 or more of the following: reCAPTCHA, an online security service, scores less than 0.50; zip codes outside of Massachusetts, Pennsylvania, or Maryland; duplicate IP addresses; and/or more than 25 surveys from the same latitude and longitude (eTable 3 in Supplement 1).^1,2,3,4,5^ For responses with 1 criterion, we reviewed the entire entry for suspicious characteristics identified in our initial recruitment stage (when we had been able to verify fraud during our interactions), such as nonsensical email addresses or being one of hundreds of surveys entered simultaneously. Analyses were conducted using SAS version 9.4 (SAS Institute).

## Results

From March 2022 to April 2023, 3561 respondents completed the eligibility screener; 3177 were fraudulent (89.2%), 228 were eligible (6.4%), and 156 were ineligible (4.4%) (Table). Fraud varied depending on the recruitment strategy in place (Figure). During initial online-only recruitment, 31 of 879 responses (3.5%) were eligible and 787 responses were fraudulent (89.5%). Switching to localized, nondigital outreach resulted in fewer responses overall, but more eligible responses (147 of 228 [64.5%]); fraudulent responses mostly occurred during the brief times when Facebook was used concurrently.

**Table.** Survey Respondents Classified as Eligible, Fraudulent, and Ineligible (N = 3561)

Classification	Respondents, No. (%)
Eligible	228 (6.4)
Ineligible (respondents did not meet inclusion criteria)	156 (4.4)
Fraudulent	3177 (89.2)
Met ≥2 criteria for fraud	2283 (64.1)
Met 1 criteria and was reviewed further for suspicious entries	894 (25.1)
No. of fraudulent respondents meeting each single type of fraud indicator^a^	
Ineligible zip codes^b^	2608 (82.1)
>25 duplicate latitude/longitude coordinates	1511 (47.6)
Duplicate IP addresses	1482 (46.7)
Online security scores <0.5	991 (31.2)
No. of participants who would have been considered eligible if fraud identification strategies were not in place	569 (16.0)

^a^ Adds up to more than 100% because fraud criteria are not mutually exclusive.

^b^ Zip codes could also be used as an indicator of ineligibility due to not meeting inclusion criteria; however the zip codes that respondents reported were well outside of where recruitment materials were posted (eg, California, Texas); thus, they were classified as fradulent.

**Figure.**
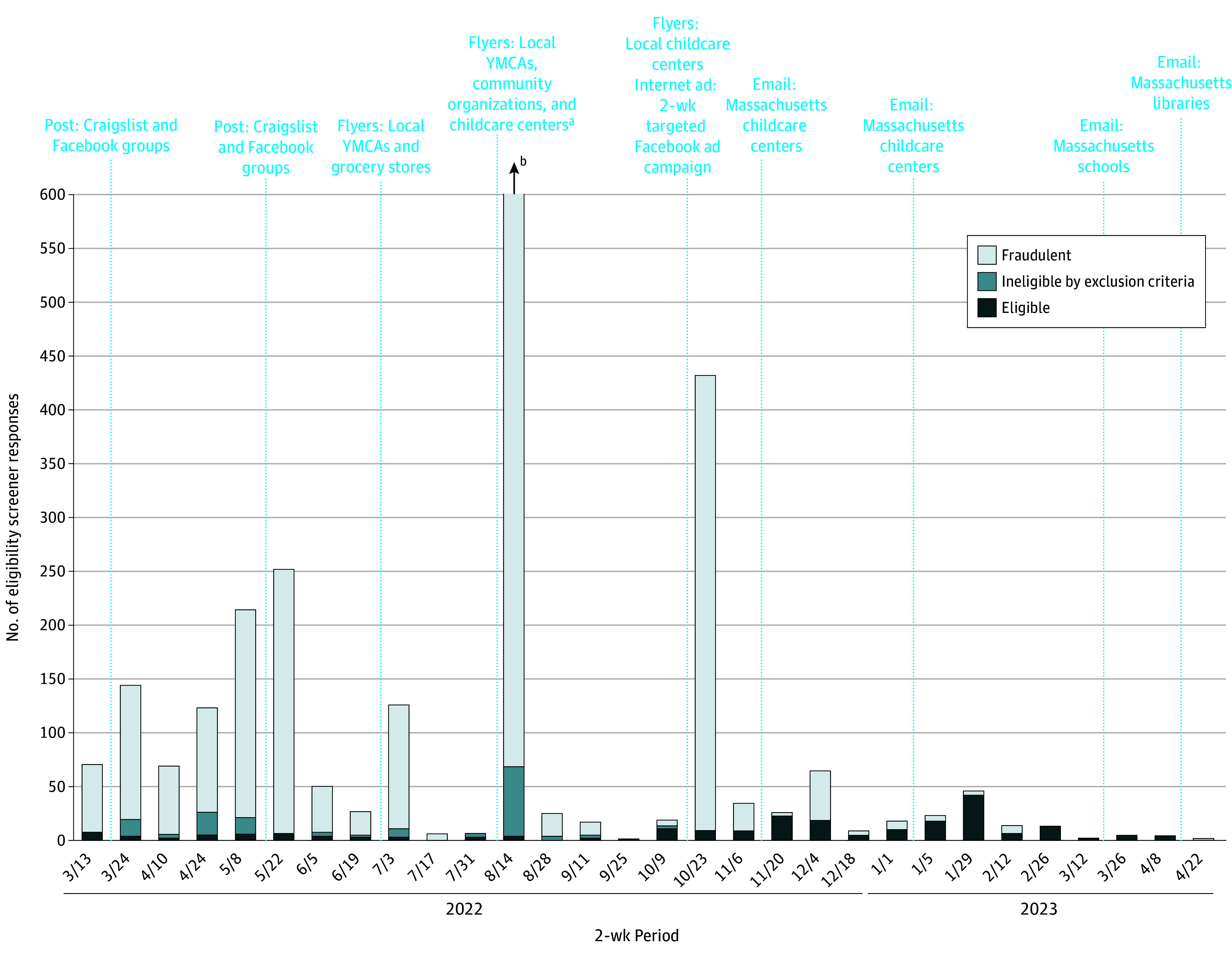
Timeline of Biweekly Recruitment Strategies With the Number of Eligible Ineligible by Exclusion Criteria, and Fraudulent Responses From March 2022 to April 2023 ^a^A childcare worker posted a study flyer to their open social media page, which led to an increase in fraudulent responses. ^b^n = 1700.

## Discussion

Our findings echo similar research, some of which has found nearly all online respondents may be fraudulent.^5,6^ Online studies, without comprehensive fraud mitigation, may be seriously flawed.^1,2,3,4,5^ While some researchers screen on the online security service scores or IP addresses alone, our results suggested this was inadequate. A study limitation is that we could not independently verify our categorizations; however, our estimated fraudulent percentages is similar to prior studies.^1,2,4,5,6^ A multilevel approach to preventing, identifying, and mitigating fraud is needed.^1,2,3,4,5^

## Notes

Supplement 1. **eTable 1.** Best Practices to Mitigate and Identify Fraudulent Activity in Survey Research **eTable 2.** Recruitment Flyers and Eligibility Screener Questions Before and After Guardrails to Identify Fraudulent Responses Were Put in Place **eTable 3.** Indicators Used in the Kids APPS Study For Identifying Fraudulent and Suspicious Activity From March 2022 to April 2023

Supplement 2.
Data Sharing Statement
