# Supplementary material for: Managing Sophisticated Fraud in Online Research
Source: JAMA Netw Open. 2025 Feb 27;8(2):e2460168. doi: 10.1001/jamanetworkopen.2024.60168 (PMC11868967; doi:10.1001/jamanetworkopen.2024.60168)
Supplement: Supplement 1. — eTable 1. Best Practices to Mitigate and Identify Fraudulent Activity in Survey Research eTable 2. Recruitment Flyers and Eligibility Screener Questions Before and After Guardrails to Identify Fraudulent Responses Were Put in Place eTable 3. Indicators Used in the Kids APPS Study For Identifying Fraudulent and Suspicious Activity From March 2022 to April 2023 [file jamanetwopen-e2460168-s001.pdf]

Supplemental Online Content

Mozaffarian RS, Norris JM, Kenney EL. Managing sophisticated fraud in online research. *JAMA Netw Open*. 2025;8(2):e2460168. doi:10.1001/jamanetworkopen.2024.60168

eTable 1. Best Practices to Mitigate and Identify Fraudulent Activity in Survey Research

eTable 2. Recruitment Flyers and Eligibility Screener Questions Before and After Guardrails to Identify Fraudulent Responses Were Put in Place

eTable 3. Indicators Used in the Kids APPS Study For Identifying Fraudulent and Suspicious Activity From March 2022 to April 2023

This supplemental material has been provided by the authors to give readers additional information about their work.

© 2025 Mozaffarian RS et al. *JAMA Network Open*.

eTable 1. Best Practices to Mitigate and Identify Fraudulent Activity in Survey Research

**1. Recruitment and incentive strategies.**
Avoid posting survey links to social media and other open web platforms (e.g., Facebook, X, Craigslist, Amazon Turk).Recruit study participants through more secure networks such as professional listservs or by collaborating with research partners.[2,3,5]
Ask research partners who help with recruitment to NOT post study materials on open social media pages or web because it could allow for fraudulent respondents to find the survey.
Do NOT include specific study inclusion criteria in recruitment materials.[2]
Include a statement in the study consent form that fraudulent participants will not be compensated.[2,4]
Use strategies to deliver incentives to eligible participants only (e.g., do not distribute incentives through automated systems; consider incentivizing through a raffle to allow time to review survey responses for fraudulent activity). Respondents demonstrating excessive interest in the incentive may be fraudulent (e.g., continuously or aggressively asking the incentive amount or when they will receive it).[2–4]
**2. Survey development**
2a. Survey development: eligibility criteria
Do not ask leading questions about eligibility criteria in screening surveys or interviews.[2]
Intentionally include options that would exclude respondents from eligibility by including invalid options[2,4].
2b. Survey development: questions to include
Include cross-survey validation questions in eligibility and study surveys to compare responses.[1,2,5]
Include open ended questions to determine if responses are coherent.[2,7,8]
Include attention checks embedded in the survey.[8]
2c. Survey development: survey platforms
Enable survey platform fraud prevention features prior to survey distribution (e.g., reCAPTCHA).[1,2,4,5,7,9]
**3. Reviewing survey data**<br>Before recruiting participants, establish which indicators below will be used to identify fraudulent and/or suspicious activity. Prior studies have set thresholds where ≥2 indicators met will be excluded as fraud. [5,6]
3a. Survey data: review survey platform metrics
Set a threshold to identify incomplete surveys which may indicate fraud (e.g., <75% complete).
Identify multiple and/or rapid re-entries from same IP addresses and/or latitude/longitude coordinates.[4]
Set a threshold to identify surveys completed in an unreasonable or improbable length of time.[2,4,5,7–10]
Identify surveys completed in a short period of time, or a suspicious time of day (e.g. 3:30am).[1,4,5,9]
Identify "all or nothing" responses (e.g., all 0's or 6's) and/or pattern responses (e.g., 1,2,3,4).[2,4,9,11,12]
3b. Survey data: review survey responses
Identify suspicious or duplicate name/email addresses (e.g., vGqa5aMBt2@gmail.com), or email addresses with a pattern FirstLastName(Numbers)@gmail.com (e.g., Johndoe123@gmail.com) [4,5,7,9–14]
Identify responses with demographic information that is different from the expected target population.[5,9]
Identify responses that demonstrate not having "insider knowledge" of the study topic that the target population should know, vague responses, and/or unable to provide details when prompted.[7,14]
Review open-ended survey questions for nonsensical or irrelevant responses; duplicate open-ended responses; or responses that are the exact duplicate of something on a website.[1,2,5,8,9,14]
Check suspicious respondent's affiliations and/or contact information by cross referencing their reported zip code, organization, and/or phone number with websites, social media, and/or White Pages.[4,10,11]
**4. System-level changes**
Journals and peer-review processes add criteria for studies using online research requiring authors to specify how bots/fraudulent responses were identified, and how many were excluded.[5]
Include recommendations in STROBE/CONSORT checklists for preventing and identifying fraud.[15,16]
IRBs provide language to researchers to include in consent forms about not providing incentives to fraudulent respondents.[5]
Train scholars and students to be aware of the impact of fraudulent participants in survey research, as well as ways to mitigate and identify such fraud.[5]

© 2025 Mozaffarian RS et al. *JAMA Network Open*.

eTable 2. Recruitment Flyers and Eligibility Screener Questions Before and After Guardrails to Identify Fraudulent Responses Were Put in Place

Before	After
**Recruitment flyers**	
If you have a child aged 2-5 years old, you may be eligible for the Kids APPS study!<br><br>You may be eligible if you are:<br>- A parent/guardian of a child aged 2-5 years old<br>- 18+ year old<br>- Living in Philadelphia, PA or Baltimore, MD<br>- Your child identifies as Black, Latinx/Hispanic, or White<br>- Your child uses a mobile device for watching shows, videos and/or playing games	Does your child play games or watch videos a tablet or smartphone? If yes, you may be eligible to participate in the Kids APPS study!<br><br>Eligible participants must be: A parent/ guardian of a young child who uses a mobile device for watching shows and/or playing games
**Eligibility Screener**	
Please enter the following information:	
Do you live in Philadelphia, PA or Baltimore, MD?<br>- Yes<br>- No	The zip code of the town you live: ______
Are you, the Parent/Guardian, 18 years old or older?<br>- Yes<br>- No	Your age: ______
Did not ask	How many children under age 8 do you have?<br>- 1<br>- 2<br>- 3<br>- 4<br>- 5+
Please select the description that best corresponds to your child's race/ethnicity:<br>- Black/African American<br>- White non-Hispanic<br>- Hispanic/Latinx<br>- Other	Is your child of Hispanic, Latinx, or Spanish origin?<br>- No, not of Hispanic, Latino, or Spanish origin,<br>- Yes, Mexican, Mexican American, Chicano, Puerto Rican, Cuban, or another Hispanic, Latino, or Spanish origin<br>- Unknown
	What is your child's race? Select all that apply.<br>- White<br>- Black/ African American<br>- American Indian or Alaska Native<br>- Asian<br>- Native Hawaiian or Pacific Islander<br>- Other
Is the child you intend to enroll in this study currently age 2,3,4, or 5?<br>- Yes<br>- No	Please select your child's age range:<br>- Under 2 years old<br>- 2-5 years old<br>- 6-8 years old

© 2025 Mozaffarian RS et al. *JAMA Network Open*.

Before	After
**Eligibility Screener**	
Please select your child's gender:<br>- Boy<br>- Girl<br>- Non-binary<br>- Prefer to self-describe	*Same, no changes*
Does your child use a mobile device (smart phone, tablet) for watching shows or videos and/or playing games?<br>- Yes<br>- No	How often does your child play games or watch shows on a mobile device (smartphone, tablet)? Does your child use a mobile device (smart phone, tablet) for watching shows or videos and/or playing games?<br>- Never<br>- 1-2 days per week<br>- More than 3 days per week
Please enter the following information:<br>- First Name (Parent/Guardian):___<br>- Last Name (Parent/Guardian):___<br>- Email:_____<br>Please enter your phone number (optional):___	*Same, no changes*
Did not ask	Where did you learn about this study?<br>- Childcare center<br>- Facebook<br>- Craigslist posting<br>- Grocery Store<br>- Flyer in YMCA or Community Center<br>- School/Afterschool program<br>- Friend/family member<br>- Other (please describe):

© 2025 Mozaffarian RS et al. *JAMA Network Open*.

eTable 3. Indicators Used in the Kids APPS Study for Identifying Fraudulent and Suspicious Activity From March 2022 to April 2023

Variable	Indication of fraudulent or suspicious response
**Eligibility Screener: Indicators of fraudulent responses**	
reCAPTCHA score[1]	ReCAPTCHA score <0.50: this technology helps determine if a response is more likely a bot or a human and is embedded in Qualtrics surveys. Scores range from 0 to 1; a score of ≥0.50 means the respondent is likely human, and score of <0.50 means the respondent is likely a bot.
Zip code	Respondents reported that they lived in a zip code outside of the targeted study location: we considered this a likely indicator of fraud given that we did not post study flyers outside of the target study area.
Duplicate IP addresses[1]	≥2 surveys were completed from the same IP address: Qualtrics collects IP addresses for each survey completed. Multiple entries from the same IP address could be an indicator of fraudulent activity because it could mean that there are multiple entries from the same computer.
Duplicate latitude/longitude coordinates[1]	>25 surveys were completed from same latitude/longitude coordinates: Multiple respondents were scanning the eligiblity screener's QR code in the same physical location where flyers had been posted (e.g. childcare programs), and therefore had the same latitude/longitude. After empirically checking the frequencies of duplicate latitude/longitude groupings, we set a threshold of >25 survey responses with duplicate coordinates as fraudulent, with the assumption that it would be extremely unlikely to have more than 25 respondents from the same location complete the survey.
**Eligibility Screener: Responses flagged as suspicious activity**	
Email addresses	Non-sensical combination of letters and numbers (e.g., vGqa5aMBt2@gmail.com); FirstNameLastNameNUMBERS@gmail.com; such emails were most often "@gmail.com" or "@outlook.com"
Date and time of survey responses	Multiple survey responses were completed over a short period of time, (e.g. 20 responses in 2 minutes).
**Study Intake Call and Data Collection:**	
Excessive interest in gift card incentive	Participants would show excessive interest in the gift card incentives: asking multiple times how much the gift card was for; asking how long after they completed the study they would receive it.
Delayed responses to questions and/or robotic-sounding voice	Researchers would ask a question and there would be a long pause; participants had a robotic-sounding voice on the phone call.
Screenshots of mobile device app usage	Screenshots that participants sent only included apps that are not traditionally used by young children (e.g., WhatsApp, Reddit); these apps were used several hours each day.

© 2025 Mozaffarian RS et al. *JAMA Network Open*.

References

1. Wang J, Calderon G, Hager ER, et al. Indentifying and preventing fraudulent responses in online public health surveys: Lessons learned during the COVID-19 pandemic. *PLOS Glob Public Heal*. 2023;3(8). doi:https://doi.org/10.1371/journal.pgph.0001452
2. Pozzar R, Hammer MJ, Underhill-Blazey M, et al. Threats of bots and other bad actors to data quality following research participant recruitment through social media: Cross-sectional questionnaire. *J Med Internet Res*. 2020;22(10). doi:10.2196/23021
3. Johnson MS, Adams VM, Byrne J. Addressing fraudulent responses in online surveys: Insights from a web-based participatory mapping study. *People Nat*. 2024;6(1):147-164. doi:10.1002/pan3.10557
4. Teitcher JEF, Bockting WO, Bauermeister JA, Hoefer CJ, Miner MH, Klitzman RL. Detecting, Prevention, and Responding to “Fraudsters” in Internet Research: Ethics and Tradeoffs. *J Law Med Ethics*. 2015;23(1):116-133. doi:10.1111/jlme.12200
5. Xu Y, Pace S, Kim J, et al. Threats to Online Surveys: Recognizing, Detecting, and Preventing Survey Bots. *Soc Work Res*. 2022;46(4):343-350. doi:10.1093/swr/svac023
6. Lawlor J, Thomas C, Drahota A. Suspicious and fraudulent online survey participation: Introducing the REAL framework. *Methodol Innov*. 2021;14(3). doi:https://doi.org/10.1177/20597991211050467
7. Kramer J, Rubin A, Coster W, et al. Strategies to address participant misrepresentation for eligibility in Web-based research. *Int J Methods Psychiatr Res*. 2014;23(1):120-129. doi:10.1002/mpr.1415
8. Webb MA, Tangney JP. Too Good to Be True: Bots and Bad Data From Mechanical Turk. *Perspect Psychol Sci*. Published online 2022. doi:10.1177/17456916221120027
9. Dewitt J, Capistrant B, Kohli N, et al. Addressing participant validity in a small internet health survey (the restore study): Protocol and recommendations for survey response validation. *JMIR Res Protoc*. 2018;20(4). doi:10.2196/resprot.7655
10. Ballard AM, Cardwell T, Young AM. Fraud detection protocol for web-based research among men who have sex with men: Development and descriptive evaluation. *JMIR Public Heal Surveill*. 2019;5(1):9-13. doi:10.2196/12344
11. Bauermeister JA, Pingel E, Zimmerman M, Couper M, Carballo-Diéguez A, Strecher VJ. Data Quality in HIV/AIDS Web-Based Surveys: Handling Invalid and Suspicious Data. *Field methods*. 2012;24(3):272-291. doi:10.1177/1525822X12443097
12. Miner MH, Bockting WO, Romine RS, Raman S. Conducting internet research with the transgender population: Reaching broad samples and collecting valid data. *Soc Sci Comput Rev*. 2012;30(2):202-211. doi:10.1177/0894439311404795
13. van Gelder MMHJ, Bretveld RW, Roeleveld N. Web-based questionnaires: The future in

© 2025 Mozaffarian RS et al. *JAMA Network Open*.

epidemiology? *Am J Epidemiol*. 2010;172(11):1292-1298. doi:10.1093/aje/kwq291

14. Roehl JM, Harland DJ. Imposter Participants: Overcoming Methodological Challenges Related to Balancing Participant Privacy with Data Quality When Using Online Recruitment and Data Collection. *Qual Rep*. 2022;27(11):2469-2485. doi:10.46743/2160-3715/2022.5475
15. Vandenbroucke JP, von Elm E, Altman DG, et al. Strengthening the Reporting of Observational Studies in Epidemiology (STROBE): Explanation and Elaboration. *Ann Intern Med*. 2007;147(8).
16. Butcher N, Monsour A, Mew E. Guidelines for Reporting Outcomes in Trial Reports The CONSORT-Outcomes 2022 Extension. *JAMA*. 2022;328(22):2252-2264. doi:10.1001/jama.2022.21022

© 2025 Mozaffarian RS et al. *JAMA Network Open*.
