# Supplementary material for: Managing Sophisticated Fraud in Online Research
Source: JAMA Netw Open. 2025 Feb 27;8(2):e2460168. doi: 10.1001/jamanetworkopen.2024.60168 (PMC11868967; doi:10.1001/jamanetworkopen.2024.60168)
Supplement: Supplement 2. — Data Sharing Statement [file jamanetwopen-e2460168-s002.pdf]

Data Sharing Statement

Data
Data available: Yes
Data types: Deidentified participant data, Data dictionary
How to access data: Data available upon request from rmozaffa@hsph.harvard.edu
When available: With publication

Supporting Documents
Document types: None

Additional Information
Who can access the data: Anyone requesting the data
Types of analyses: Any purpose
Mechanisms of data availability: Without investigator support
Any additional restrictions: None
